# Th2-T_RMs_ Maintain Life-Long Allergic Memory in Experimental Asthma in Mice

**DOI:** 10.3389/fimmu.2019.00840

**Published:** 2019-04-24

**Authors:** Berislav Bošnjak, Sahar Kazemi, Lukas M. Altenburger, Gordana Mokrović, Michelle M. Epstein

**Affiliations:** Experimental Allergy Laboratory, Department of Dermatology, Medical University of Vienna, Vienna, Austria

**Keywords:** T_RMs_, Th2 cells, asthma, allergy, mice

## Abstract

Allergic asthma is a chronic inflammatory remitting-relapsing disease affecting the airways. Long-lived allergen-specific memory CD4^+^ T helper 2 (Th2) cells in mice persist in lungs for more than 2 years after the induction of experimental allergic asthma (EAA). To further understand lung Th2 memory cells, we tracked CD4^+^ T cells in spleen and lungs from healthy mice, through the initiation of acute EAA, recovery (remission), and allergen-induced disease relapse. We identified a lung CD3^+^CD4^+^ cell subset that expresses CD44^hi^CD62L^−^CD69^+^ST2^+^, produces Th2 cytokines, and mediates allergen-induced disease relapse despite treatment with FTY720 and anti-CD4 antibody. These cells reside in the lung tissue for the lifetime of mice (>665 days) and represent long-lived pathogenic Th2 tissue resident memory cells (T_RMs_) that maintain “allergic memory” in lung. We speculate that these data implicate that human Th2-T_RMs_ sentinels in lungs of patients are poised to rapidly respond to inhaled allergen and induce asthma attacks and that therapeutic approaches targeting these cells may provide relief to patients with allergic asthma.

## Introduction

Individuals who are susceptible to allergic disease or are atopic may react to certain inhaled allergens like pollen or house dust mite by developing allergic asthma (1–3), which is a chronic inflammatory disease affecting the airways and is characterized by immune responses resulting in reversible airway obstruction and potential structural lung damage (4, 5). Both in humans and in experimental animals, CD4^+^ T-helper (Th) lymphocytes central to the pathogenesis of allergic asthma have been extensively studied and, yet, the underlying mechanisms of this relapsing-remitting disease remain incompletely understood. To further dissect the role of Th cells, we established an allergen-induced relapsing-remitting experimental mouse model that mimics seasonal or intermittent bronchial asthma brought on by allergen sensitization and repeated exposure to the sensitizing allergen (6). In this model, long-lived allergen-specific memory CD4^+^ Th2 cells are present within cellular infiltrates in the lungs for the lifetime of the mouse, which corroborates findings in asthmatic patients who have Th2 cells in sputum, bronchoalveolar lavage fluid (BAL), and lung biopsies both during and out of allergy season (7, 8). These observations either suggest that these Th2 memory cells recirculate through or persist in the lungs of patients and mice.

In mice recovered from their first episode of allergen-induced experimental asthma, there are quiescent Th2 memory cells within infiltrates near small, medium, and large airways (6). These T cells respond to inhaled allergen and then lead to disease relapse that mimics a seasonal human asthma attack with eosinophilic airway inflammation, mucus hypersecretion, and airway hyperresponsiveness (AHR) (9). Although these long-lived lung Th2 memory cells are considered pathogenic, they have not been fully characterized. In recent years, tissue resident memory cells (T_RMs_) have been found in a variety of peripheral tissues including lungs and can be distinguished from circulating memory cells by certain cell surface expression markers (CD69, CD103), their inability to freely circulate, and their resistance to intravenous anti-CD4 monoclonal antibody (mAb) treatment (10–17).

We speculate that mice with allergic asthma have allergen-specific Th2-T_RMs_ residing in the lungs and acting as sentinels responding to allergen exposure and driving allergen-induced exacerbations of asthma, which is probably similar in asthmatic patients. The support of this phenomenon in patients is evidence that asthmatics have Th2 cells in their lungs (7, 8), healthy lungs have memory CD4^+^ and CD8^+^ T cells expressing T_RM_ markers, e.g., CD69 and CD103 (18–21), patients with HLA–mismatched lung transplants have donor T_RMs_ for more than 1 year after transplant (22), and children with allergic asthma improve when allergen is removed from their environment, only to relapse upon re-exposure to allergen (23). Here, we sought to explore the importance of T_RMs_ in allergic asthma using a mouse model of relapsing-remitting experimental allergic asthma (EAA).

## Materials and Methods

### Mice

Female BALB/c mice from Charles River (Sulzfeld, Germany) were kept in specific pathogen-free conditions and provided ovalbumin (OVA)-free food (SSNIFF, Soest, Germany) and autoclaved tap water *ad libitum*. In all experiments, mice were age-matched and were 6–8 weeks old at the time they were first immunized.

### Antigen Sensitization and Induction of Allergic Asthma and Relapse

Mice were immunized and challenged with allergen to induce acute EAA followed by recovery and relapse and compared to healthy mice and sensitized, unchallenged mice (Supplementary Figure 1A). One cohort of mice was analyzed for airway and lung inflammation, mucus production, and serum antibody, and the distinct phases of disease are characterized in Supplementary Figures 1B–E. To sensitize mice, they received two intraperitoneal (i.p.) injections of 10 μg of OVA (grade V, Sigma-Aldrich, MI) dissolved in 200 μl Dulbecco's phosphate buffered saline (PBS) on days 0 and 21 (*sensitization*). To initiate disease, we exposed sensitized mice to nebulized OVA in PBS (1%) with an ultrasonic nebulizer (Aerodyne, Kendall, Neustadt, Germany) for 60 min twice daily on days 28 and 29 (*initiation*). Disease initiation was analyzed for cell populations at 3, 7, 14, and 35 days after the last aerosol challenge, or mice were allowed to recover for a minimum of 100 days (*recovery* ≥ days 100–636). *Relapse* was induced in recovered mice with a single intranasal (i.n.) OVA challenge (100 μg) in 50 μl of PBS with light anesthesia and mice were then analyzed for cell populations at days 3, 7, 14, and 35. Recovered mice were randomly chosen at different times from disease initiation (see figure legends) for sample collection and/or rechallenge.

### *In vivo* CD4^+^ T Cell Depletion and Antibody Labeling

In sensitized and recovered mice, we injected LEAF™ purified anti-mouse CD4 mAb GK1.5 or LEAF™ purified rat IgG2b, κ isotype control antibody (0.2 mg, BioLegend, San Diego, CA) i.p. on three consecutive days. Seventy-two hours later, we administered APC-labeled anti-CD4 mAb (2.5 μg; clone RM4-4) intravenously (i.v.). After 10–15 min, lungs and spleens were resected and lungs were perfused via the pulmonary artery with 15 ml PBS containing 2% fetal bovine serum (FBS; Invitrogen, Carlsbad, CA) until they were white. The lungs were then cut into small pieces and digested with 150U of collagenase I (Invitrogen) and 50U of DNase I (Sigma-Aldrich) in RPMI (Invitrogen) containing 5% FBS for 45 min at 37°C. The resulting single cell suspension was homogenized using a 15 ml glass homogenizer (Kimble Chase, Vineland, NJ) and centrifuged at 200 × g. Splenocytes were isolated from minced spleens and suspended in PBS with 2% FBS. Cell suspensions were treated with FACS Lysing Solution (BD Bioscience, San Jose, CA) to remove erythrocytes and washed with PBS with 2% FBS before FACS staining or use in cell assays.

### *In vitro* Stimulation of Lung and Spleen Cells

Lung and spleen cells were incubated in RPMI with 5% FBS at 37°C overnight. We then added thioglycolate-elicited peritoneal macrophages labeled with cell proliferation dye eFluor450 (eBioscience, San Diego, CA) and pulsed with OVA or bovine serum albumin (BSA, 20 μg/ml; Sigma) overnight. Three hours later, we added protein transport inhibitor cocktail (eBioscience) and incubated for another 6 h. As a positive control, cells were stimulated with the eBioscience cell stimulation cocktail plus protein transport inhibitors for 6 h before they were collected for FACS staining.

### *In vivo* FTY720 Treatment

Recovered BALB/c mice were treated with 250 μl (0.5 mg/kg) FTY720 (Fingolimod, Calbiochem, San Diego, CA) i.p. dissolved in distilled water or vehicle alone daily for 3 consecutive days and assessed 1 day later.

### Fluorescence-Labeled Antibodies for Flow Cytometry

The following antibodies were used for FACS: PerCP-labeled CD4 (clone RM4-5), APC-labeled CD4 (clone RM4-4), Pacific Blue-labeled CD62L (clone MEL-14), Alexa Fluor 700-labeled CD44 (clone IM7), and Brilliant Violet 510-labeled CD69 (clone H1.2F3) mAbs from Biolegend. PE-labeled CD3 (clone 145-2C11; BD Biosciences), FITC-labeled ST2 (clone DJ8) (MD Bioproducts Zürich, Switzerland). PE-labeled CCR7 (clone 17A2), APC-labeled IL-4 (clone 11B11), PE-labeled IL-5 (clone TRFK5), eFlour 450-labeled IFNγ (clone XMG1.2), e-Fluor 450-labeled IL-13 (clone eBio13A), and APC-labeled IL-17 (clone eBio17B7) from eBioscience.

### Flow Cytometry

Single cell suspensions from lung and spleen were blocked with 6 μg of normal mouse and rat IgG antibodies (Invitrogen) and then incubated with the noncompeting anti-CD4 mAb clone RM4-5 and other fluorochrome-labeled antibodies against extracellular markers at 4°C for 30 min. Fluorescence minus one (FMO) controls were used, if required. After washing, cells were stained with eFluor-780 fixable viability dye (eBioscience). Data acquisition was performed on a BD LSRFortessa cell analyzer (BD Bioscience) with 7-color detection and at least 300,000 (lung) or 50,000 (spleen) total events collected. Analysis was done with FlowJo 9.6 (Tree Star Inc., San Carlos, CA). The gating strategy for CD4^+^ T cell populations in the non-autofluorescent live cell gate is shown in Supplementary Figure 3. After staining with extracellular markers and viability dye, the cells were fixed and permeabilized using an intracellular fixation and permeabilization buffer set (eBioscience) according to the manufacturer's recommended protocol. The cells were then incubated overnight at 4°C followed by a 45 min incubation with anti-cytokine antibodies at RT. Cells were then washed with permeabilization buffer, followed by PBS with 2% FBS. The gating strategy for CD4^+^ T cell populations in the non-autofluorescent live cell gate is shown in Supplementary Figure 3. The total number of cells in a population was calculated using total number of live cells counted with the cytometer and FACS data as described previously (24). Briefly, the percentage of each cell population was expressed as the percentage of total live cells (total cells), and the number of cells in that population was calculated using the following formula: cell number (populationX) = Total number x populationX (% of total cells)/100%.

### Immunofluorescence Microscopy

Mice were administered APC-labeled anti-CD4 mAb (2.5 μg, clone GK1.5, Biolegend) i.v. Lungs were harvested 10–15 min later, inflated with a mixture of OCT and PBS (1:1), and embedded in OCT. Frozen tissues were cut into 9 μm sections at −20°C and stored at −80°C until further use. Frozen sections were rehydrated in PBS and non-specific binding sites were blocked with 5% BSA in PBS for 30 min. To detect protected CD4^+^ T cells, sections were stained with Alexa Fluor 594-labeled anti-CD4 (GK1.5, Biolegend) overnight at 4°C. Sections were then washed with PBS, stained with DAPI (Invitrogen) for 3 min and mounted with Fluoroshield Mounting Medium (Abcam, Cambridge, UK). Images were acquired within 3 days with an automated Leica DM6000B microscope (Wetzlar, Germany), Baumer TXG50c camera (Friedberg, Germany), HC Plan APO 20x/0.7 + HC PL FLUOTAR 5x 0.15 Leica objectives. Photomicrographs were analyzed with TissueFAXS© software (TissueGnostics, Vienna, Austria) and ImageJ (NIH, Bethesda, MD).

### Airway Inflammation

BAL was done at the indicated times after the last OVA-challenge in anesthetized mice by intubating and flushing the airways 3 times with PBS for a total volume of 1 ml. The total number of cells in BAL was enumerated in a Neubauer hemocytometer and differential cell count was done by morphological examination of >300 cells on Kwik-Diff (Thermo Fisher Scientific Inc., Pittsburgh, PA)-stained cytospin slides. Eosinophil, lymphocyte, neutrophil and macrophage counts were calculated by multiplying the total BAL cell count with cell percentages.

### Lung Inflammation and Mucus Secretion

Following BAL, lungs were fixed in 4% buffered formalin and embedded in paraffin. Lung sections (3 μm) containing main stem bronchi were stained with hematoxylin and eosin (H&E) and graded using 2 scores done for inflammation intensity and the extent of inflammation within the lung. For intensity: 0–no inflammatory infiltrates; 1–occasional cells or cuffs of cells around bronchi/vessels; 2–thin layer of inflammatory cells (1–2 cells) around bronchi/vessels; and 3–thick layer of inflammatory cells (>2 cells) around bronchioles/vessels. For extent of inflammation: 0–no inflammatory infiltrates; 1–inflammatory infiltrates in central airways; 2–inflammatory infiltrates extending to the middle third of the lung parenchyma; and 3–inflammatory infiltrates extending to the periphery of the lungs. A histological score was calculated for each lung lobe as the product of intensity and extent of inflammation, and averaged for each sample. Adjacent lung sections were stained for eosinophils using the Luna stain. Eosinophils were counted on 10 random fields (40x magnification) containing alveoli and without major airways/vessels (selected at low power magnification), and averaged for each lung. Lung sections were stained with periodic acid-Schiff (PAS) and enumerated for mucus-containing cells/mm of basement membrane and averaged for each mouse. All analyses were done on a BX40 microscope using 10x, 20x, 40x, and 100x objectives (Olympus Europa Holding GmbH, Hamburg, Germany) with a Progress Speed XT^Core^ 5 camera using ProgRes®CapturePro 2.9.0.1 software (both Jenoptik, Jena, Germany).

### Statistical Analysis

All data are presented as mean ± SEM and were analyzed using GraphPad Prism v.5.0 (GraphPad Software Inc., San Diego, CA) with Student *t*-tests, ANOVA, for the number of eosinophils in the airways and on lung sections and the number of mucus-producing cells in the central airways: One-way ANOVA followed by Tukey's multiple comparison test, and for inflammation scores: Chi-square test. Values of *p* < 0.05 were considered significant.

## Results

### CD3^+^CD4^+^ T Cells Expand and Contract in the Lungs at Distinct Phases of EAA

EAA was induced in mice by i.p. OVA administration (sensitization), followed by aerosol challenge leading to the onset of acute disease (initiation). The mice were then left to recover in the absence of allergen for 100–636 days (recovery), and then rechallenged with the same allergen, which led to a robust disease exacerbation (relapse) (Supplementary Figure 1A). The course of EAA was monitored by assessing the inflammatory response in the lungs and allergen-specific antibodies in serum. Disease initiation and relapse were characterized by eosinophilic lung inflammation, mucus hypersecretion, serum OVA-specific IgG1 and IgE, while during recovery, eosinophilia, mucus, and IgE were absent (Supplementary Figures 1B–E).

To follow CD4^+^ T lymphocyte populations over the course of disease beginning with healthy (naïve) mice, we utilized an *in vivo*–*in vitro* antibody labeling method to quantify CD3^+^CD4^+^ Th cells in the lung and compared these to spleen. To specifically distinguish *in vivo* CD4-labeled from unlabeled CD4^+^ T cells, we injected mice i.v. with anti-CD4 mAb (clone RM4-4), removed the cells 10 min later and stained them *in vitro* with a different anti-CD4 mAb (clone RM4-5). Using this technique, it was possible to discriminate between “labeled or circulating” and “protected” cells (unlabeled/unstained by i.v. anti-CD4 mAb) because of preferential labeling of cells in circulation compared with cells that are lodged in the tissue (Supplementary Figures 3, 4A). To characterize CD3^+^CD4^+^ T cells over the course of allergic asthma, we evaluated lungs and spleens of (1) healthy mice, (2) sensitized, unchallenged mice, (3) during initiation at days 3, 7, 14, and 35 after the last aerosol challenge, (4) during recovery, and (5) at relapse on days 3, 7, 14, and 35 after the last challenge using flow cytometry (Supplementary Figure 2).

**Figure 1:**
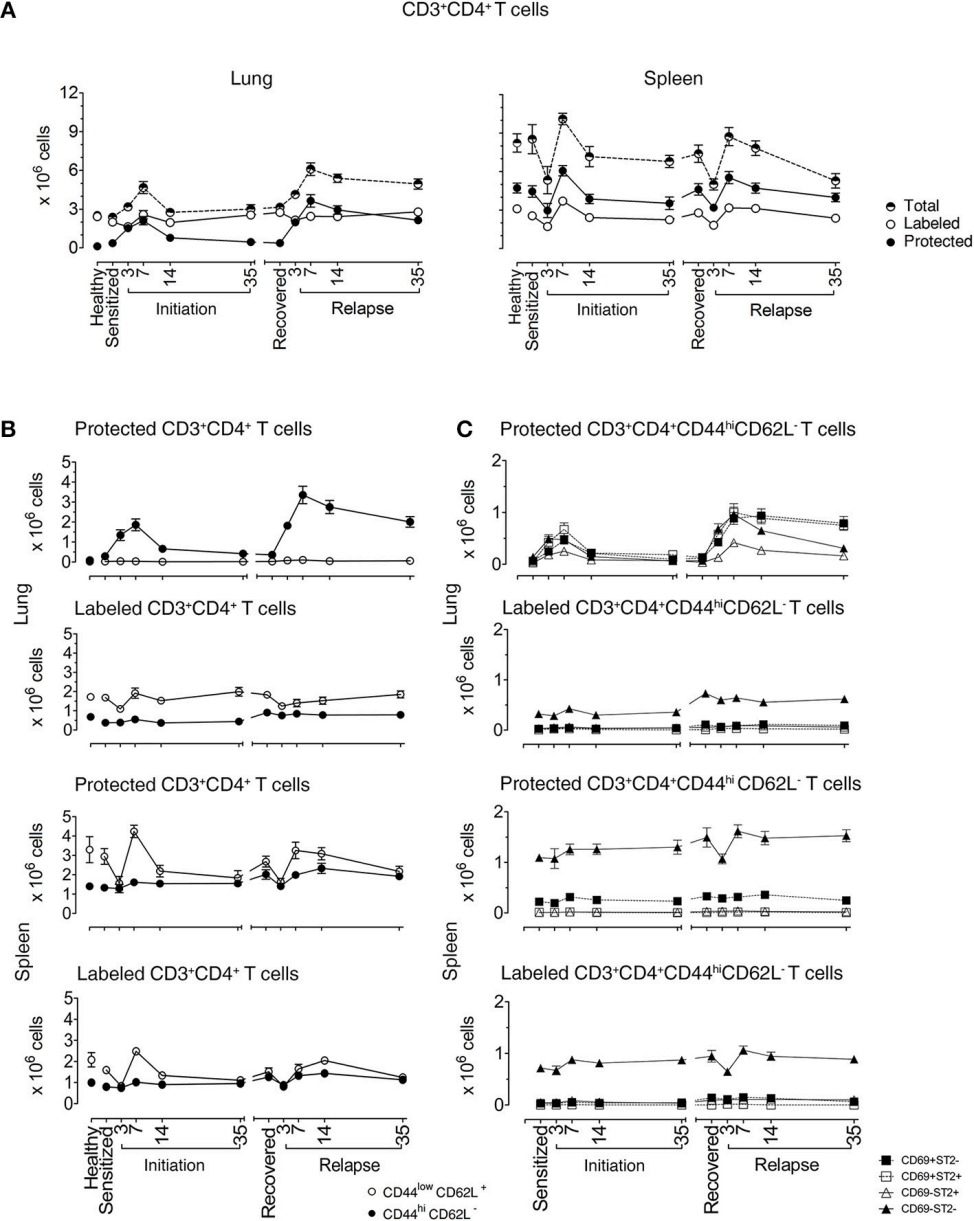
Lung CD3^+^CD4^+^ T cells are protected from *in vivo* antibody labeling during allergic asthma in mice. **(A)** Healthy mice and mice over the course of allergic asthma were *in vivo* labeled with anti-CD4 mAb (clone RM4-4) and organs were extracted 10–15 min later. Lung and spleen cell suspensions were prepared and stained with a non-overlapping anti-CD4 mAb (clone RM4-5) and antibodies for other extracellular markers for flow cytometric analysis. Number of total, protected and labeled CD3^+^CD4^+^ T cells in the lungs and spleens are shown during initiation, after 185 days of recovery (range from 142 to 234 days) and during relapse. **(B)** Number of protected and labeled effector/memory and naïve Th cells in lungs and spleens of healthy animals and mice in various stages of allergic asthma. **(C)** Number of protected and labeled CD3^+^CD4^+^CD44^hi^CD62L^−^ T cells with differing ST2 and CD69 expression in spleen and lung of healthy controls, during asthma initiation, after 185 days of recovery (range from D142–234) and relapse. Data are shown as mean ± SEM of 8–16 mice/time point and were compiled from 6 independent experiments.

In the lungs of healthy mice, we distinguished labeled and protected CD3^+^CD4^+^ T cell baseline numbers at 2.42 ± 0.11 × 10^6^ and 0.12 ± 0.01 × 10^6^, respectively. In sensitized mice, there were similar numbers of labeled cells (2 ± 0.22 × 10^6^), but there were 3-fold more protected cells (0.36 ± 0.15 × 10^6^) illustrating that protected CD3^+^CD4^+^ T cells are generated in the lung just following i.p. allergen without respiratory tract challenge (25). Labeled cells remained constant at each phase of disease with a range of 2.0 × 10^6^–4.30 × 10^6^. In contrast, the numbers of protected cells varied significantly over the course of disease. In mice challenged with aerosolized OVA, protected cells increased and peaked at D7 (2.10 ± 0.31 × 10^6^ cells) and slowly decreased to D35 (0.45 ± 0.10 × 10^6^ cells), though they remained significantly elevated compared with healthy mice. During recovery, protected CD3^+^CD4^+^ T cells (0.37 ± 0.05 × 10^6^ cells) were stable for over 100 days, but upon secondary aerosol OVA rechallenge, the numbers increased and peaked again on D7 (3.641 ± 0.47 × 10^6^ cells) and decreased by D35 after relapse (2.145 ± 0.25 × 10^6^ cells), but were approximately 4-fold higher than D35 after initiation. These data show that protected CD3^+^CD4^+^ T cells expanded and contracted during the course of EAA (Figure 1A).

In the spleen, total CD3^+^CD4^+^ T cell numbers were higher than in lungs and there were more protected compared to labeled cells. Both labeled and protected cells were influenced by the phase of disease and had a different kinetic pattern compared with the lung. There was a reduction in cell numbers up to D3, followed by an increase that peaked at D7 during both initiation and relapse (Figure 1A). Notably, on D3 during the induction of disease initiation and relapse, inhaled allergen increased CD4^+^ T cells in lung while simultaneously decreasing CD4^+^ T cells in spleen. These data show that inhaled allergen influenced local and systemic CD4^+^ T cell numbers and behavior.

### Protected Memory CD4^+^ T Cells in Lungs During Disease Recovery Are T_RMs_

We first characterized the naïve and memory phenotype of labeled and protected CD4^+^ T cells, by following the expression of CD44 and CD62L markers on these cells in lungs and spleens throughout the course of disease. Figure 1B illustrates the numbers and percentages (Supplementary Figures 4B,C) of labeled and protected naïve (CD4^+^CD44^lo^CD62L^+^) and memory/effector (CD4^+^CD44^hi^CD62L^−^) cells. Labeled cells were predominantly naïve in lung and spleen, while protected cells were memory/effector cells in lung and naïve cells in spleen.

Secondly, we examined the kinetics of protected and labeled CD3^+^CD4^+^CD44^hi^CD62L^−^ T memory/effector cells expressing CD69 (10, 11, 15, 26–28) and ST2 (24), which are markers for T_RMs_ and Th2 cells, respectively (Figure 1C). The major change in the kinetics of the response occurs in protected cells in the lungs which mirrors the expansion and contraction of the CD3^+^CD4^+^CD44^hi^CD62L^−^ T memory/effector cell population seen in Figure 1B, regardless of CD69 and ST2 expression. The CD69^+^ST2^+^, CD69^−^ST2^−^, and CD69^+^ST2^−^ subpopulations are well represented compared to the CD69^−^ST2^+^ T cell population. Notably, in the labeled lung memory/effector cell population, there are more CD69^−^ST2^−^ cells compared with the other subpopulations with similar, but less dramatic kinetics. The most prominent protected and labeled spleen memory/effector subpopulation lacked expression of CD69 and ST2 and varied according to disease phase, whereas all other subpopulations remained constant.

**Figure 2:**
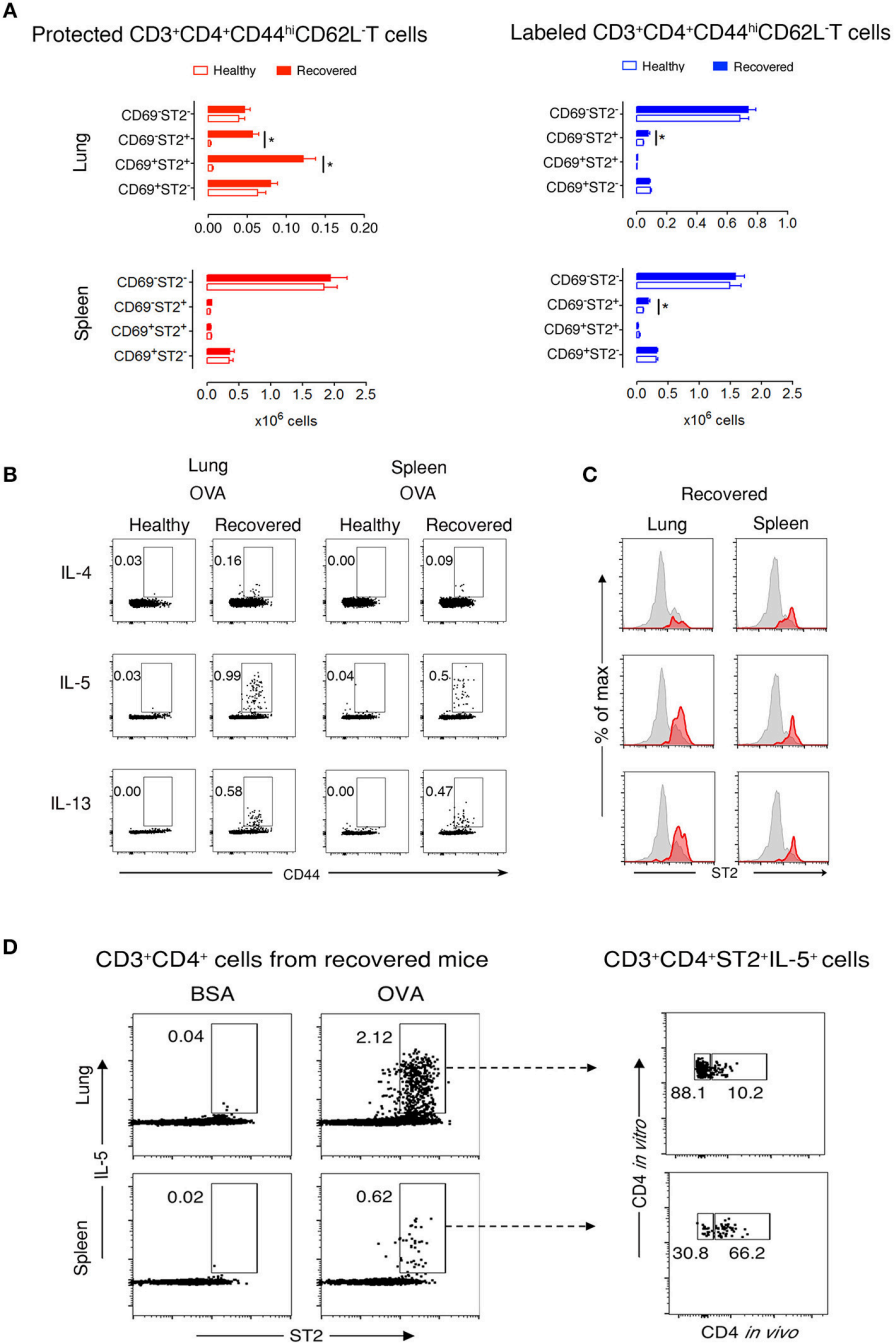
Protected allergen-specific lung CD3^+^CD4^+^ T cells express CD69 and ST2. **(A)** Absolute numbers of protected and labeled CD3^+^CD4^+^CD44^hi^CD62L^−^ T cells differing in CD69 and ST2 expression in lungs and spleens of mice after an average of 199 days (range from D164-244) of recovery and age-matched healthy controls. Data are shown as mean ± SEM of 12 mice/time point and were compiled from three independent experiments. ^*^p < 0.05 using two-way ANOVA. **(B)** Representative dot-plots indicating that allergen-specific Th2 cells are present in lungs and spleens of recovered mice. At D227, lung and spleen cells from healthy and recovered mice (*n* = 3) were isolated, pooled, and stimulated for 9 h with OVA-loaded peritoneal macrophages. Dot-plots show CD44 vs. intracellular IL-4, IL-5, or IL-13 on gated CD3^+^CD4^+^ cells. Numbers indicate the percentage of cells in the respective cytokine-producing gate. Data are representative of three independent experiments. **(C)** Histograms show ST2 expression on total CD3^+^CD4^+^CD44^hi^ T cells (gray) and IL-4-, IL-5-, or IL-13-producing CD3^+^CD4^+^CD44^hi^ T cells (red) in lungs and spleens of recovered mice (*n* = *3*) at D227. Data are representative of three independent experiments. **(D)** Lung and spleen cells from recovered mice (*n* = *3*) at D227 were isolated 10–15 min after i.v. anti-CD4 mAb administration, pooled, and stimulated for 9 h with BSA- or OVA-loaded peritoneal macrophages. Dot-plots on the left side show ST2 vs. intracellular IL-5 on gated CD3^+^CD4^+^ cells. Dot plots on the right side indicate that the majority of IL-5 producing OVA-specific CD3^+^CD4^+^ ST2^+^cells in the lung, but not in the spleen, are protected from *in vivo* antibody labeling. Numbers indicate the percentage of cells in the respective cytokine-producing gate. Data are representative of three independent experiments.

Next, we focused on lung and spleen cells from recovered and healthy mice to further characterize memory T cell subpopulations. During recovery, CD44^hi^CD62L^−^ T cells are memory but not effector cells. Thus, determining CD69 and ST2 cell marker expression would define T_RM_ Th2 cells in the protected and labeled CD3^+^CD4^+^CD44^hi^CD62L^−^ T cell populations. We observed two abundant protected cell populations of CD69^+^ST2^+^ and CD69^−^ST2^+^ memory T cells in recovered compared to healthy lungs (Figure 2A and Supplementary Figure 5). In contrast, labeled lung T cells were predominantly CD69^−^ST2^−^ memory T cells with a significant, but low increase in the number of CD69^−^ST2^+^ memory cells in recovered vs. healthy lungs. In spleen, there were no significant differences detected between healthy and recovered mice for protected cells stained with CD69 and ST2, whereas labeled spleen cells were similar to labeled lung cells. Notably, CD103, another accepted marker for T_RMs_, expression was negative on lung resident cells (data not shown). Taken together, these data demonstrate that the protected CD3^+^CD4^+^CD44^hi^CD62L^−^CD69^−/+^ST2^+^ T cells in the lung phenotypically appear to be T_RMs_.

### Protected CD3^+^CD4^+^ T Cells at Recovery Are OVA-Specific

To determine whether CD3^+^CD4^+^ T cells are OVA-specific, we stimulated lung and spleen cells *in vitro* with OVA or non-crossreactive BSA. We found that recovered compared with healthy CD3^+^CD4^+^CD44^hi^ T cells produced Th2 cytokines in response to OVA, but not to BSA, and that neither population produced IFNγ nor IL-17α (Figure 2B and Supplementary Figure 6). Moreover, a majority of IL-5- and IL-13-expressing cells in the lung co-expressed ST2 (Figure 2C). Remarkably, these recovered CD3^+^CD4^+^ST2^+^ Th2 cells had a secondary OVA-specific *in vitro* response 636 days after disease initiation (Supplementary Figure 7), demonstrating that the cells are maintained following one episode of EAA for a lifetime. Figure 2D illustrates that 88% of OVA-specific CD3^+^CD4^+^ST2^+^IL-5^+^ T cells in recovered mice are protected in the lung compared to 30% in spleen.

### Lung T_RMs_ do Not Circulate

**Figure 3:**
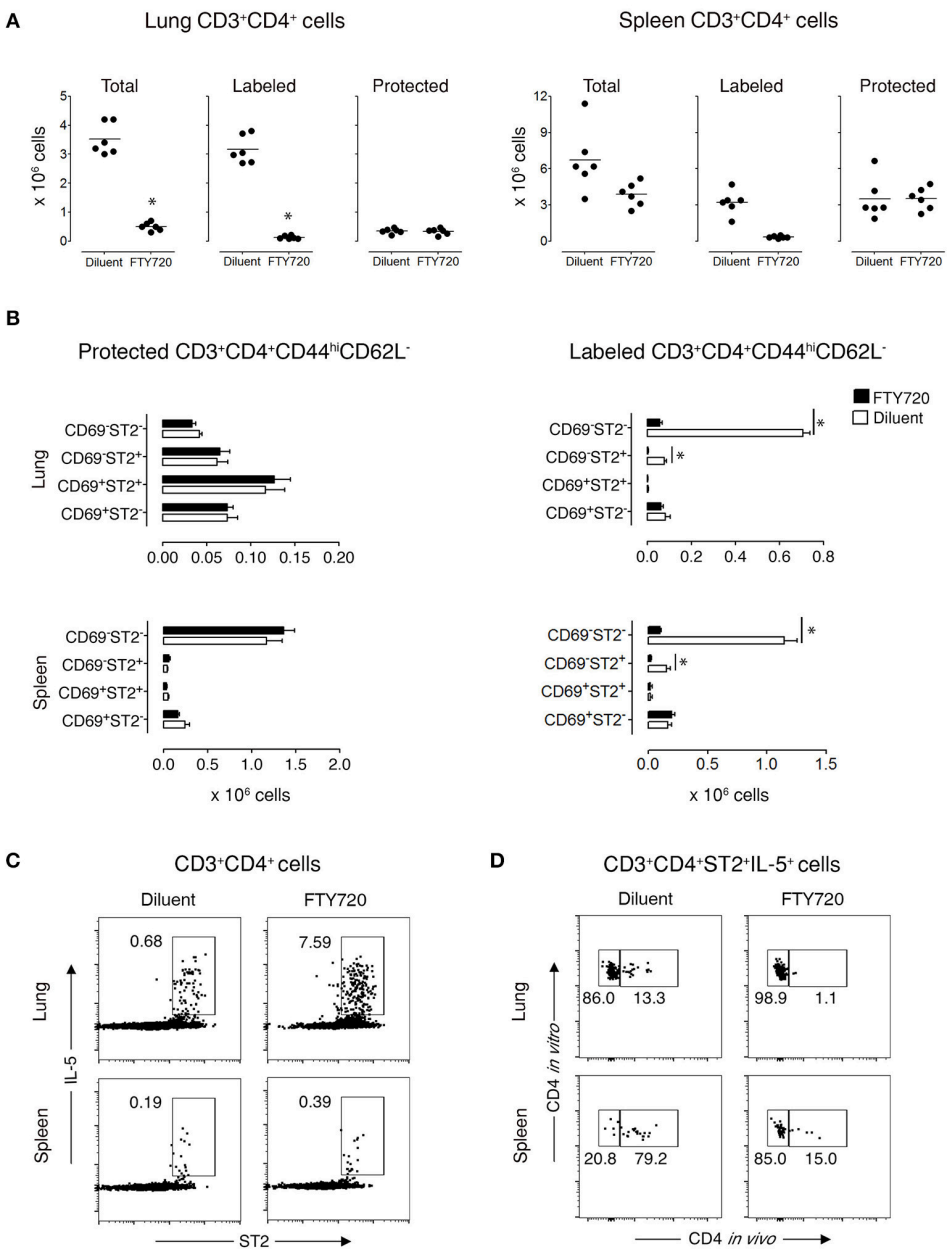
Protected allergen-specific CD3^+^CD4^+^ T cells remain in the lung and spleen after FTY720 treatment. Mice at D172 and D174 of recovery from disease initiation were treated with FTY720 (0.5 mg/kg) or diluent i.p. daily for three consecutive days. One day later, mice were assessed 10-15 min after i.v. injection of anti-CD4 mAb to determine the effect of treatment on labeled and protected CD3^+^CD4^+^ T cell populations in lung and spleen. **(A)** Number of total, labeled, and protected CD3^+^CD4^+^ T cells in lungs and spleens of control- and FTY720-treated groups. Data are presented as individual values (dots) and group means (lines) compiled from 2 independent experiments (*n* = *6*). ^*^p < 0.05 using Student's *t*-test. **(B)** Number of protected and labeled CD3^+^CD4^+^CD44^hi^CD62L^−^ expressing surface markers CD69 and ST2 in spleens and lungs of diluent- and FTY720-treated mice. Data are presented as mean ± SEM compiled from two independent experiments (*n* = *6*). ^*^p < 0.05 using two-way ANOVA. **(C)** Representative dot-plots indicating that allergen-specific Th2 cells remain in the lungs and spleens of recovered mice after FTY720 treatment. Lung and spleen cells from three mice/group were isolated, pooled, and stimulated for 9 h with OVA-loaded peritoneal macrophages. Dot-plots show ST2 vs. intracellular IL-5 on gated CD3^+^CD4^+^ cells. Numbers indicate the percentage of cells in the respective cytokine-producing gate. Data are representative of two independent experiments. **(D)** Dot-plots show frequency of labeled and protected IL-5-producing CD3^+^CD4^+^ST2^+^ T cells upon stimulation with OVA-loaded peritoneal macrophages. Data are representative of two independent experiments.

To demonstrate that protected lung T cells reside in the lungs and are not temporarily passing through the lung parenchyma, we treated recovered mice for 3 days with diluent or FTY720, which blocks lymphocyte emigration from lymph nodes (29, 30). FTY720 treatment markedly reduced the total Th cell population in the lungs, which was entirely due to diminished circulation of labeled cells by 92% (Figure 3A and Supplementary Figures 8A,C), with a significant reduction in 2 subpopulations of labeled CD3^+^CD4^+^CD44^hi^CD62L^−^CD69^−^ST2^−^ and CD3^+^CD4^+^CD44^hi^CD62L^−^CD69^−^ST2^+^ T cells by 92 and 95%, respectively, but no change in any of the protected memory Th cell subpopulations (Figure 3B and Supplementary Figure 8B). In spleen, the effect of FTY720 was less dramatic with an 84% reduction of total Th cells accounted for by a significant reduction in labeled CD3^+^CD4^+^CD44^hi^CD62L^−^CD69^−^ST2^−/+^ T cell subpopulations (Figure 3B and Supplementary Figure 8B). Similar to the lungs, the protected splenic Th memory cells remained constant (Figure 3B). Furthermore, the remaining protected CD3^+^CD4^+^ST2^+^ Th cells were capable of producing OVA-specific IL-5 *in vitro* (Figures 3C,D). These data show that functional, allergen-specific protected CD4^+^ T cells following FTY720 treatment are maintained, which indicates that they are resident OVA-specific Th2 memory cells/Th2-T_RMs_.

### T_RMs_ Mediate Disease Relapse

**Figure 4:**
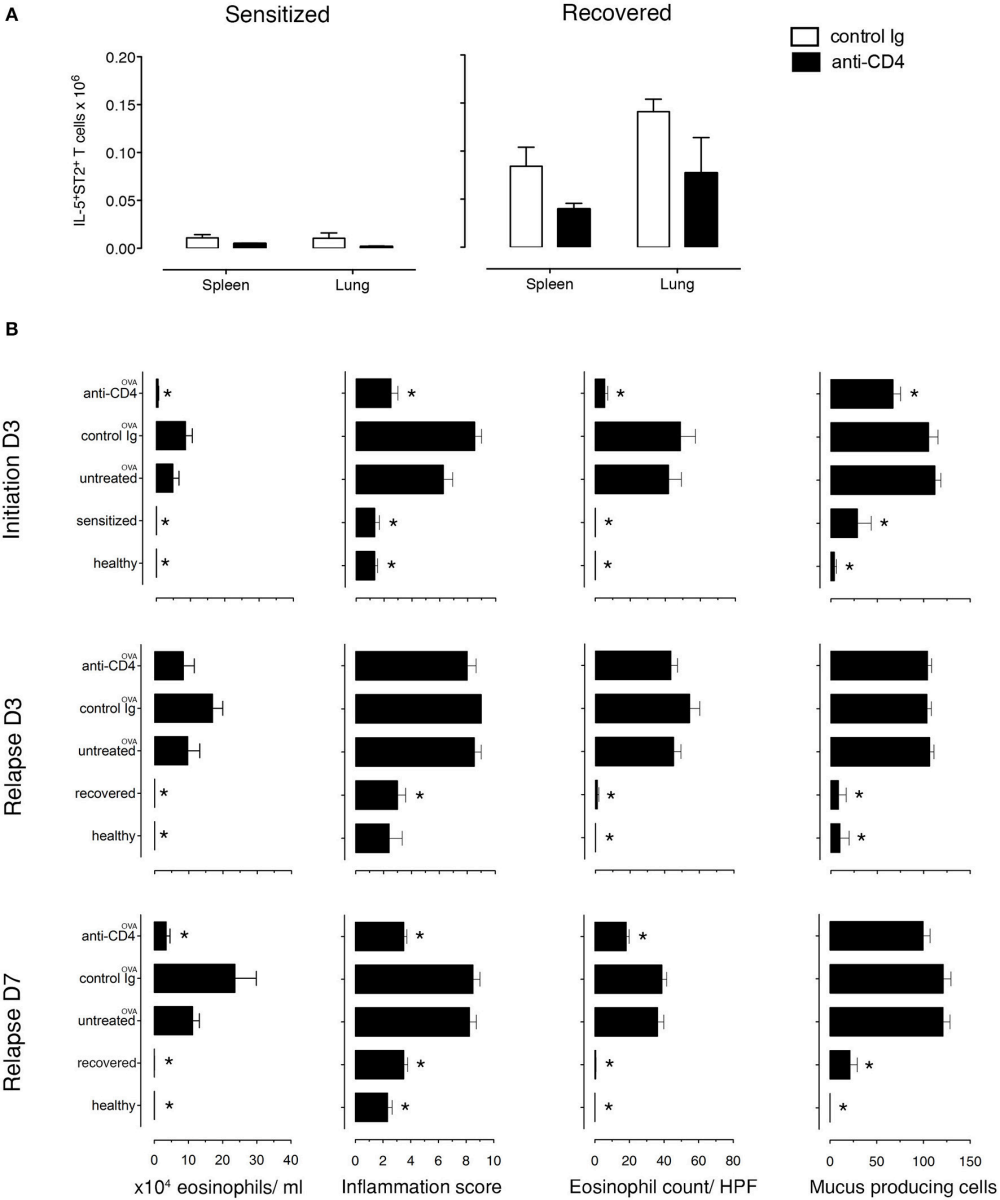
Lung allergen-specific Th2 cells remaining after anti-CD4 mAb depletion mediate early inflammatory response to allergen rechallenge. Sensitized and recovered mice were treated i.p. with 0.2 mg of anti-CD4 depleting mAb (clone GK1.5; anti-CD4) or isotype control antibody (control Ig) for 3 consecutive days. Three days after the last treatment, single lung and spleen cell suspensions were prepared for FACS analysis for one cohort of mice and a second cohort of mice was challenged with 100 μg of OVA i.n. to induce allergic asthma in sensitized mice (untreated ^OVA^, control Ig ^OVA^, anti-CD4 ^OVA^) or disease relapse in recovered mice (untreated ^OVA^, control Ig ^OVA^, anti-CD4 ^OVA^). Disease parameters were evaluated at D3 and D7 after allergen challenge. **(A)** Lung and spleen cells from sensitized or recovered mice (*n* = *6–8*) treated with control Ig or anti-CD4 mAb were stimulated for 9 h with OVA-loaded peritoneal macrophages. Bar graphs indicate IL-5^+^ST2^+^ T cell numbers presented as mean ± SEM from two independent experiments. **(B)** Absolute eosinophil numbers in BAL grade of inflammation in H&E-stained lung sections, eosinophil numbers on LUNA-stained lung sections, and the number of mucus-containing cells in central airways on PAS-stained lung sections during initiation, at D3 and D7. Data are presented as mean ± SEM from two independent experiments (*n* = *6–8*). ^*^p < 0.05 is statistically significant compared with the untreated group.

Because T_RMs_ are largely resistant to mAb-induced depletion, anti-CD4 mAb treatment mainly depletes circulating cells (31–33). To determine the role of T_RMs_ in disease induction, we treated recovered and sensitized mice i.p. with either anti-CD4 mAb (GK1.5) or rat IgG2b, κ isotype control antibody for 3 days. We found that anti-CD4 depleted the majority of CD3^+^CD4^+^ T cells in the lungs (96.5% in sensitized and 81.75% in recovered) and in the spleens (99.5% in sensitized and 99.75% in recovered) (Supplementary Figure 9A). Moreover, all depleted T cells were CD4^+^ labeled *in vivo* (Supplementary Figure 9B) and all IL-5^+^, *in vivo* CD4^+^-labeled T cells were eliminated from both organs (Supplementary Figure 9C). Notably, populations of IL-5^+^ T cells in lungs (4.65%) and in spleens (<1%) of recovered mice were not labeled with *in vivo* CD4 mAb and were present in numbers <0.1% in sensitized mice (Supplementary Figure 9C). We then established the number of antigen-specific anti-CD4 mAb-resistant (IL-5^+^ST2^+^) T cells and found that recovered mice have more than sensitized mice by 8.2-fold in the spleen and 46-fold in the lung. Moreover, anti-CD4 mAb treatment decreased the numbers in both lungs and spleen of recovered and sensitized mice leaving 70,000 anti-CD4 mAb-resistant antigen-specific (IL-5^+^ST2^+^) T cells in the lungs and 39,000 in spleens of recovered mice and 1,500 in lungs and 4,700 in spleens of sensitized mice (Figure 4A).

Importantly, to determine whether the anti-CD4 mAb-resistant cells mediated disease relapse, we assessed disease parameters following anti-CD4 mAb treatment and OVA-rechallenge. Sensitized and recovered mice receiving control rat mAb and OVA had the expected increase in eosinophilic airway and lung inflammation and mucus hypersecretion compared to sensitized and recovered mice. However, sensitized mice treated with anti-CD4 and OVA (during initiation) compared with untreated mice had an 88% reduction of airway eosinophils, a 40.2% reduction of mucus produced from goblet cells, a drop in lung tissue eosinophil numbers by 87.1%, and lung tissue inflammation score from 6.2 to 2.1. In contrast, anti-CD4 and OVA treatment in recovered mice had no effect on airway and lung eosinophilia or mucus production compared with untreated mice at D3, indicating that disease relapse was not affected by anti-CD4 treatment. However, disease parameters were reduced by D7 showing that in our model, T_RMs_ are responsible for early, but not late responses (Figure 4B and Supplementary Figures 10, 11).

## Discussion

The relapsing-remitting nature of seasonal (e.g., pollen) or intermittent (e.g., cat allergens) allergic disease strongly supports a role for immunological memory allergen-specific Th2 cells. We previously determined that long-lived allergen-specific Th2 cells rapidly induce disease relapses upon exposure to allergen in sensitized mice and reside in the lungs throughout the lifetime (>800 days) of the mouse after only one episode of EAA (6). Here, we investigated circulating and resident CD4^+^ Th cell subpopulations during the course of disease. We show that long-lived allergen-specific Th2 memory cells in the lungs are “allergic memory” Th2-T_RMs_. Moreover, allergic Th2-T_RMs_ in lung are crucial for the rapid initiation of allergen-induced disease relapses mimicking allergic asthma attacks in patients.

We tracked CD4^+^ T cells in anti-CD4 experiments at disease initiation and relapse and determined that circulating CD4^+^ T cells and lung T_RMs_ are essential at different times during disease. Firstly, administration of anti-CD4 mAb reduced the initiation of allergic lung disease by eliminating the predominant population of circulating CD4^+^ Th cells generated during sensitization. These data support previous studies by confirming that systemic (i.p.) allergen sensitization and boost generates mostly circulating cells with few T_RMs_ due to the lack of localized allergen exposure (17). Secondly, anti-CD4 treatment in recovered mice rechallenged with allergen did not reduce the severity of disease relapse compared with untreated controls as evaluated at D3. This indicates that there are sufficient numbers of Th2 memory cells or, more specifically, Th2-T_RMs_ present in the lung that are capable of rapidly responding to allergen. These findings confirm studies showing that T_RMs_ in lungs of mice in infectious models play a role in the early response to viral infection (34–36). Thirdly, while our data demonstrate a critical role of lung Th2-T_RMs_ in relapsing EAA early after allergen rechallenge (evaluated at D3), circulating CD4^+^ T cells appear to contribute to the full extent of disease relapse. At D7 after allergen rechallenge, there is significantly less severe disease in response to allergen compared with untreated controls. These data suggest that the circulating T cells eliminated by anti-CD4 are required later to immigrate to lung and expand the effector CD4^+^ T cell population to either enhance or prolong the overall tissue response. Taken together, these experiments demonstrate distinct roles for circulating CD4^+^ Th cells and lung Th2-T_RMs_ despite the possibility that a minor population of transient circulating cells migrated through the lung at the time of *in vivo* labeling (37) remained unlabeled.

To further elucidate a role for CD4^+^ Th subpopulations, we tracked T_RMs_ in lung and followed naïve and memory resident and circulating cells in lung and spleen. In health, there are mostly naïve circulating CD4^+^ Th cells with few resident cells found in lung. As disease develops (at initiation), there is a substantial increase in the numbers of protected cells in the lungs, which are likely Th effector cells. Most of these effector cells vanish within ~1 month with few resident memory Th cells or T_RMs_ remaining. At recovery, there are more T_RMs_ than in healthy lungs confirming the residence of these cells and indicating that they are poised and prepared to respond immediately to allergen and initiate an asthma attack (relapse). Indeed, at D3 after OVA-rechallenge, resident cells increase in the lungs as they react against the allergen. These findings in mice resemble the clinical situation in sensitized atopic patients who rapidly respond to allergen (e.g., pollen or cat allergen) re-encounter. In addition to the changing population of lung resident cells, there is a large number of circulating CD4^+^ T cells in the spleen that also change during the course of disease. These circulating cells decrease, both at the early stage of disease initiation and at relapse (up to D3) followed by an increase by D7. The mechanism underlying the concomitant inverse proportion of circulating splenic CD4^+^ T and lung resident cells during disease initiation and relapse might be explained by circulating splenic CD4^+^ T cells migrating from spleen as CD4^+^ T effector cells to lung where they clonally expand T_RMs_. Alternatively, circulating CD4^+^ T cells from the spleen are non-specific bystander cells that enhance the ability of the T_RMs_ to expand within the lungs. There are additional subsets including naïve protected CD4^+^ T cells that are probably in white pulp of the spleen, which was previously shown (15, 38) and naïve circulating CD4^+^ T cells in lung that remain constant during all stages of disease. The role of either of these populations is not clear, but it is probable that naïve circulating cells in the lungs represent non-specific bystander cells.

To determine the phenotype of CD4^+^ T subpopulations of circulating and resident cells, we focused on naïve, memory, CD69 and ST2 marker expression, with the latter two considered markers for T_RMs_ and Th2 cells, respectively (10, 11, 15, 24, 26–28). Recovered lungs have increased numbers of CD4^+^CD44^hi^CD69^+^ST2^+^ and CD4^+^CD44^hi^CD69^−^ST2^+^ resident memory T cells compared to healthy lungs. These cells are Th2-T_RMs_ regardless of the expression of CD69, which indicates that CD69 might not be the most accurate marker for T_RMs_, which confirms previous studies (39, 40). Upon allergen challenge of the respiratory tract, the majority of allergen-specific memory Th2 cells remain in the lungs as T_RMs_, which we speculate reside there to ensure faster and more effective responses upon allergen encounter. Notably, a small population of memory Th2 cells (10 times lower than the lung population) persists in the spleen that probably contains central memory and secondary lymphoid organ T_RMs_ (40–43). It is conceivable that these cells, though low in number, participate in the maintenance of memory, are a potential source of memory cells, or contribute to the expansion of the memory/memory effector cell populations upon encounter with allergen. The exact role for these Th2 spleen cells has yet to be elucidated.

To further characterize the CD4^+^ T subpopulations of circulating and resident cells, we focused on the class of the response. In addition to the expression of ST2, T_RMs_ produced allergen-specific Th2 cytokines. However, in contrast to a previous study in an allergic house dust mite (HDM) model, allergen-specific Th1 or Th17 cytokines were not produced by spleen and lung cells (17), which is likely related to the combination of HDM proteins compared to a single purified OVA protein. Although there is an absence of allergen-specific non-Th2 cytokines, stimulation with PMA induced IFNγ and IL-17α from lungs and IFNγ from spleen cells of healthy and recovered mice showing a predominant OVA-specific Th2 response and an apparent presence of other bystander cells in the tissue.

Strong evidence for the presence of lung resident memory cells was derived from FTY720 administration, which reduced circulating CD3^+^CD4^+^CD44^hi^CD62L^−^CD69^−^ST2^−/+^ (lung>spleen) and increased the percentage of OVA-specific CD3^+^CD4^+^CD44^hi^CD62L^−^ST2^+^ T cells in the lung. These findings demonstrate that persistence of lung T_RMs_ relates to their inability to circulate and emigrate from lung tissue. T_RMs_ remain in lung and have no need to migrate, as previously observed (15, 28, 44). Furthermore, the maintenance of lung T_RMs_ occurs in the absence of other circulating or lymphoid T cell immigration to lung, which is supported by previous studies (15, 28). Our data show that cells resisting FTY720 and anti-CD4 are retained in the lungs and mediate disease, which agrees with other studies in mice (17, 28) and clinical studies demonstrating little benefit of anti-CD4 treatment in severe asthma (45, 46).

Our data focus on the allergic response in the lungs to foreign protein. However, these results are generalizable, as there are additional studies addressing T_RMs_ with similar properties in allergy, allergic lung disease, and viral disease (15–17, 28, 47). Our results with OVA confirm previous studies showing that T_RMs_ are involved in the pathogenesis of allergic asthma induced with HDM (17). However, these studies addressed memory responses after a 60–80 day recovery period (17, 28), whereas our study is the first to show that T_RMs_ are maintained for over 600 days and proves that once memory is established in lungs following a single episode of EAA, the animals maintain memory cells for their lifetime. This has clinical implications including an explanation for why the majority of allergic asthma patients remain allergic throughout their lifetime. However, it is likely that repeated allergen exposures will continuously maintain and/or expand T_RMs_ over time leading to potentially more severe asthma. It is also likely that homeostatic mechanisms that normally limit the numbers of T cells, will protect against an overabundance of antigen-specific T_RMs_ in the tissue. In contrast, it is difficult to explain why some infants with allergic asthma “grow out” of their allergy, but this might be a consequence of an immature immune system which generates only a few T_RMs_ or an uncooperative microenvironment in the peripheral tissues that does not maintain them. In children who do possess T_RMs_ in one peripheral organ, it is tempting to speculate that T_RMs_ could underlie the “allergic march” by extension to other organ systems. However, this might relate to circulating memory cells, as there are no studies examining the duration of both allergen-specific T_RM_ and circulating memory cells though short-term parabiosis experiments show that T_RMs_ do not migrate from the tissue (28).

In summary, animals recovered from only one episode of allergic disease harbor lung T_RMs_ throughout life as sentinels for the rapid initiation of allergen-induced disease relapses is clinically relevant. This model provides an opportunity to investigate novel treatment approaches for allergic disease focusing on allergen-specific quiescent T_RMs_ during recovery and activated T_RMs_ during relapse as therapeutic targets.

## Ethics Statement

This study was carried out in strict accordance with the guidelines for the care and use of laboratory animals of the Austrian Ministry of Science. The protocol was approved by the Committee on the Ethics of the Austrian Ministry of Science (Number: GZ: 66.009/0330-II/3b/2013). All painful procedures were performed under anesthesia, and all efforts were made to minimize suffering.

## Author Contributions

BB designed and performed experiments, analyzed the samples and contributed to the manuscript preparation. SK designed and performed experiments, analyzed the samples and contributed to the manuscript preparation. LA performed experiments and contributed to the manuscript preparation. GM contributed to immunofluorescent staining of the lungs. ME supervised experiments and contributed to the manuscript preparation. All authors read and approved final version of the manuscript.

### Conflict of Interest Statement

The authors declare that the research was conducted in the absence of any commercial or financial relationships that could be construed as a potential conflict of interest.

## Abbreviations

EAA: experimental allergic asthma
T_RMs_: Tissue resident memory cells
Th2: T helper type 2
CD: Cluster of differentiation
AHR: Airway hyperresponsiveness
BAL: Bronchoalveolar lavage fluid
i.p.: Intraperitoneal
i.n.: Intranasal
i.v.: intravenous
mAb: monoclonal antibody
OVA: Ovalbumin
PMA: Phorbol 12-myristate 13-acetate
BSA: Bovine serum albumin
PAS: Periodic acid Schiff
H&E: Hematoxylin and eosin.
