# Supplementary material for: Th2-TRMs Maintain Life-Long Allergic Memory in Experimental Asthma in Mice
Source: Front Immunol. 2019 Apr 24;10:840. doi: 10.3389/fimmu.2019.00840 (PMC6493194; doi:10.3389/fimmu.2019.00840)
Supplement: Supplementary file 1 [file Data_Sheet_1.pdf]

Supplementary Material

1 Supplementary Figures

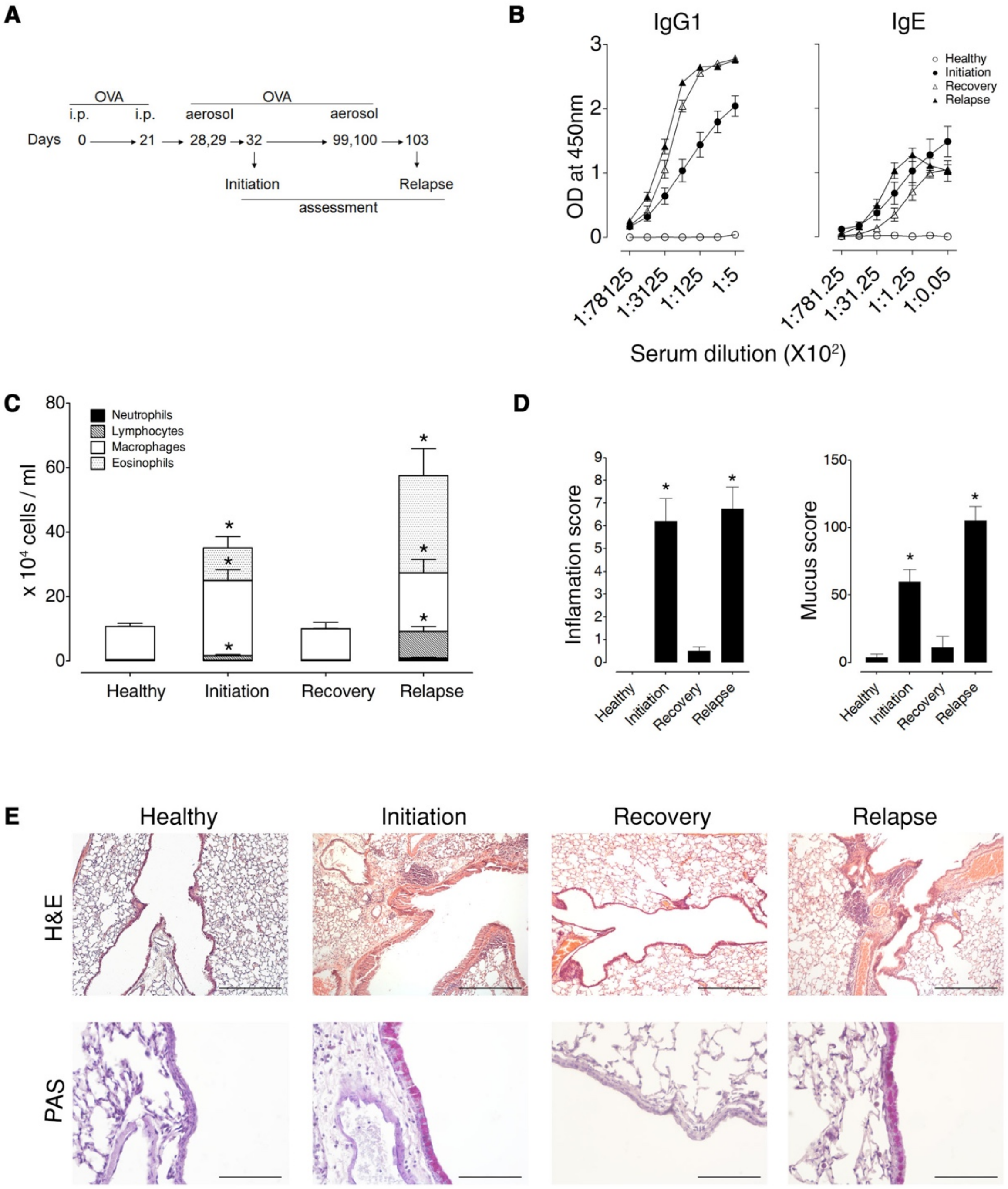

Supplementary Figure 1 | Distinct phases of allergic asthma in mice. **(A)** Schematic presentation of the EAA protocol. **(B)** Serum OVA-specific IgG1 and IgE titres. **(C)** Total BAL cell numbers and differential counts/ml of BAL from healthy mice, at initiation (D32), during recovery and asthma relapse (D103). **(D)** Grade of lung inflammation (left) and number of mucus-positive cells/mm basement membrane (right). (B-D) Data presented as mean ± SEM and are representative of 2 independent experiments (*n=8-10*). One-way ANOVA (*p < 0.05). **(E)** Representative photomicrographs of H&E- (upper row) and PAS- (lower row) stained lung tissue sections. On H&E-stained lung sections, areas of highest-density inflammation are shown. On PAS-stained lung sections, fuchsia-stained mucus in epithelial goblet cells were detected during asthma initiation and relapse, but were not present in healthy mice and during recovery. Scale bar is 400µm for H&E and 100 µm for PAS-stained sections.

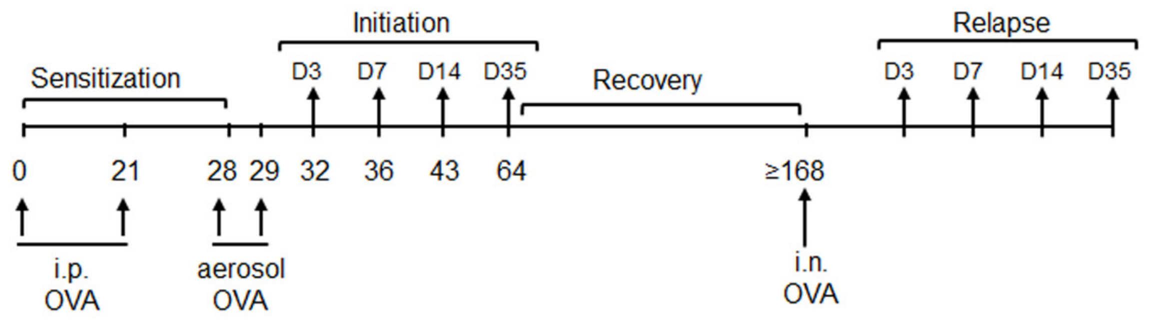

Supplementary Figure 2 | Scheme of experimental model for allergic asthma initiation, recovery and relapse in mice used in all experiments.

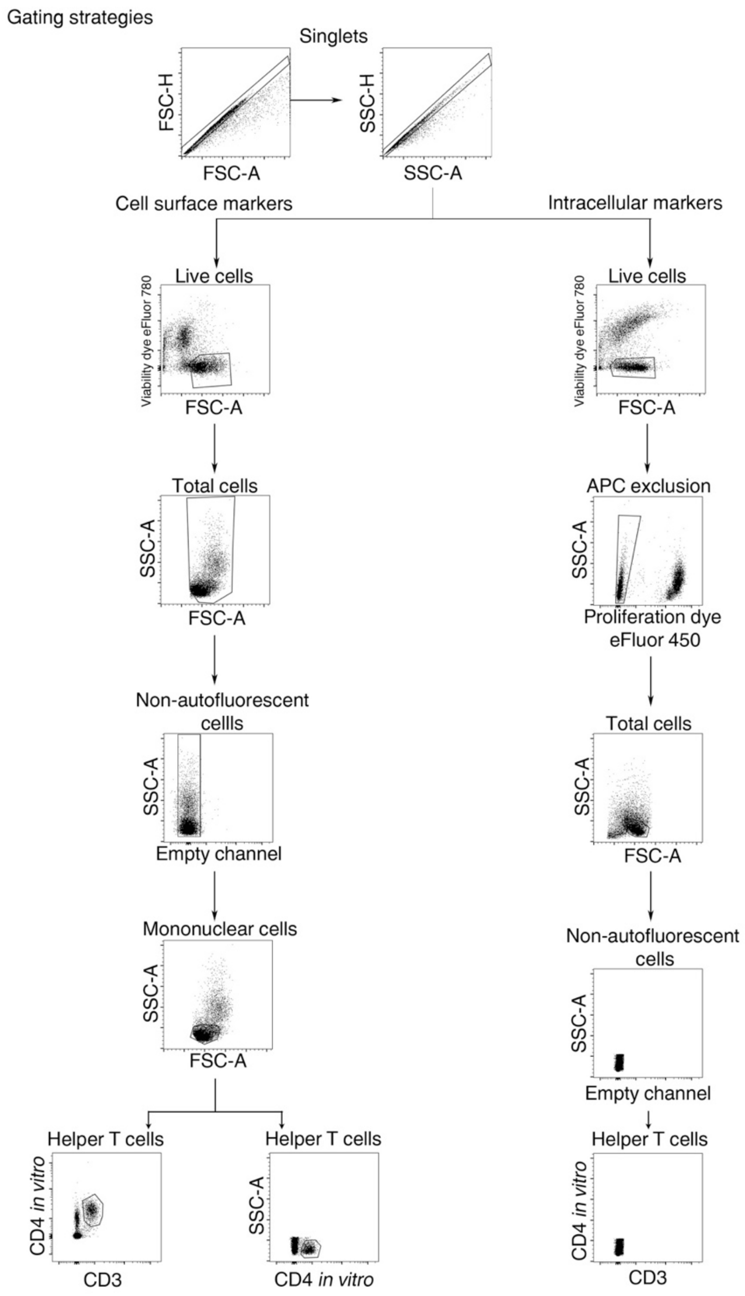

Supplementary Figure 3 | Gating strategies. Representative dot-plots from lung cells of recovered mice showing the gating strategies to identify Th cells for cell surface marker labeling and intracellular cytokine detection. The same gating strategy was used for splenocytes.

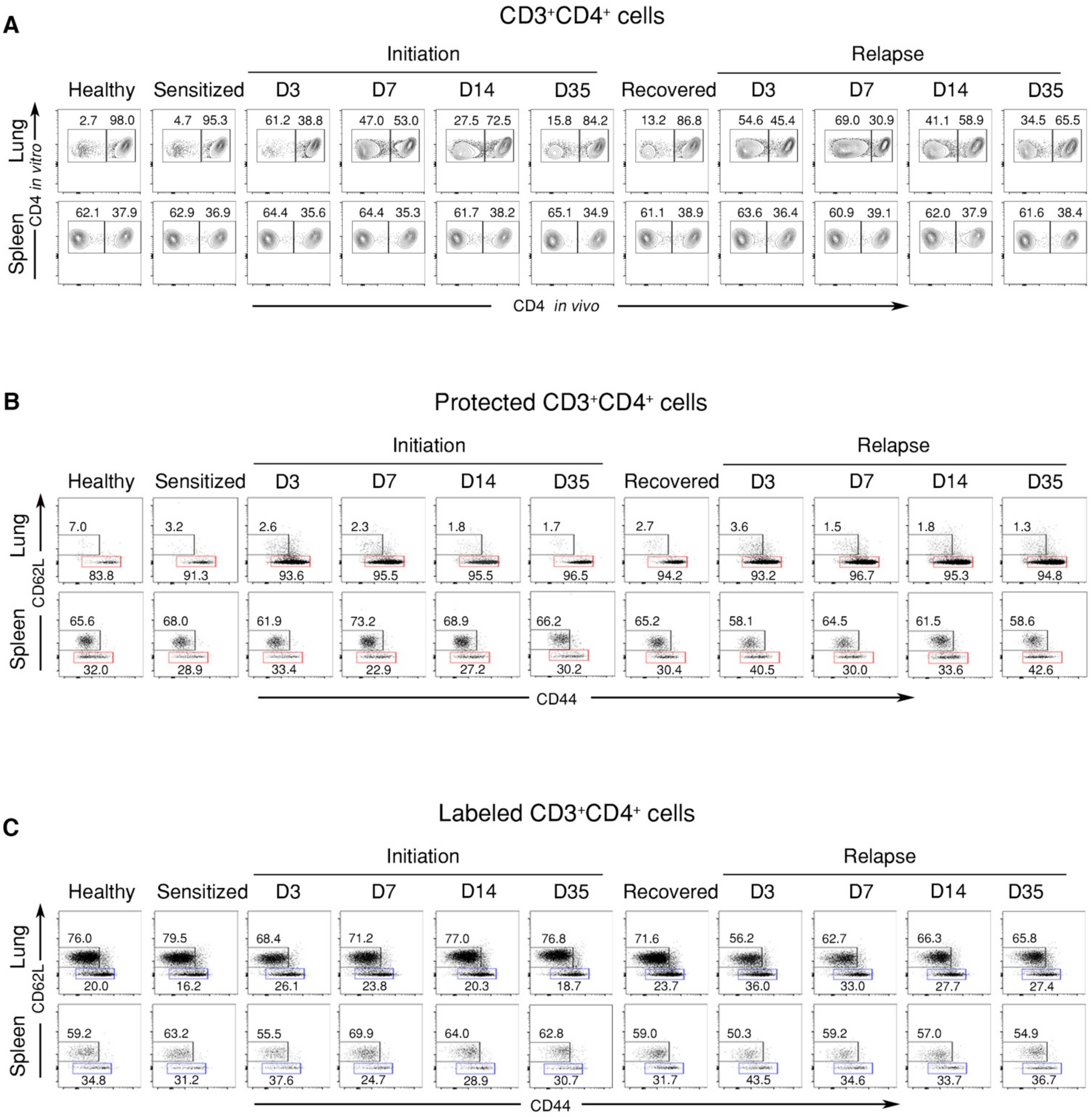

Supplementary Figure 4 | Representative flow cytometry plots of Figure 1. Anti-CD4 mAb clone RM4-4 was administered i.v. and 10-15 min later organs were extracted and cell suspensions prepared, and stained with a non-overlapping anti-CD4 mAb (clone RM4-5) and antibodies for other extracellular markers for FACS analysis. **(A)** Representative contour-plots of $CD3^{+}CD4^{+}$ T cells labeled with or protected from i.v. anti-CD4 mAb. **(B)** Representative dot-plots of CD62L and CD44 expression on protected $CD3^{+}CD4^{+}$ T cells from spleen and lung. **(C)** Representative dot-plots of CD62L and CD44 expression on labeled $CD3^{+}CD4^{+}$ T cells from spleen and lung.

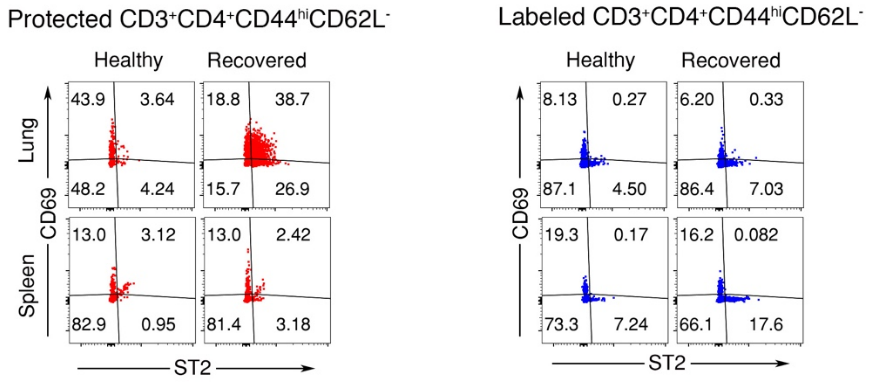

Supplementary Figure 5 | Protected lung $CD3^+CD4^+CD44^{hi}CD62L^-$ T cells of recovered mice express CD69 and ST2**.** Anti-CD4 mAb clone RM4-4 was administered i.v. and 10-15 min later organs were extracted and cell suspensions prepared, and stained with a non-overlapping anti-CD4 mAb (clone RM4-5) and antibodies for other extracellular markers for FACS analysis. Representative dot-plots of ST2 and CD69 expression on protected and labeled $CD3^+CD4^+CD44^{hi}CD62L^-$ T cells in spleen and lung of healthy controls and mice recovered from allergic asthma initiation for 185 days (D213).

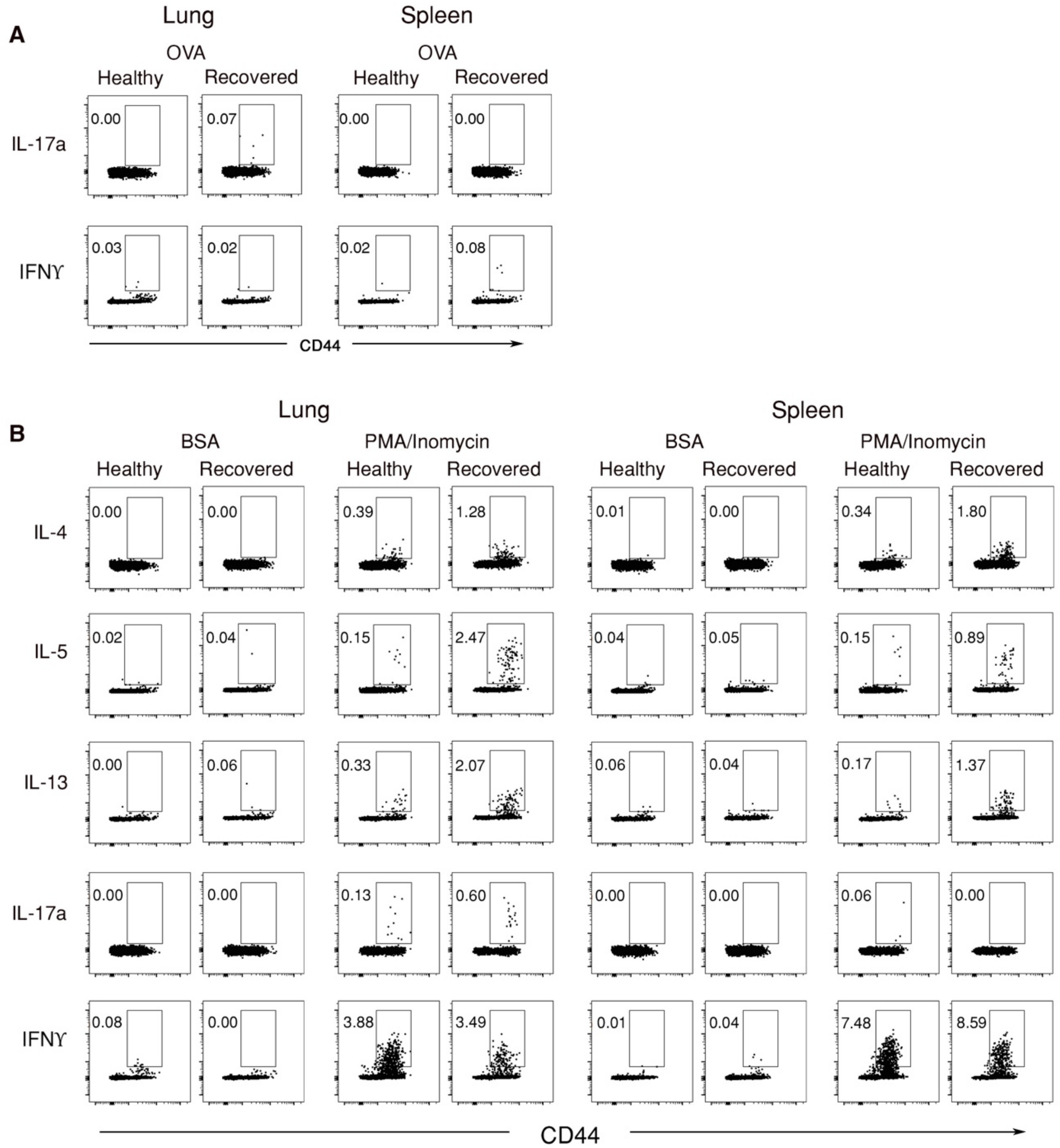

Supplementary Figure 6 | Intracellular cytokine expression of CD3$^+$CD4$^+$ memory T cells from lungs and spleen cells of healthy and recovered mice. Lung and spleen cell suspensions from healthy or recovered mice (3 mice/group) at D227 were prepared, pooled and stimulated for 9 h with OVA- or BSA-loaded peritoneal macrophages or combination of PMA and ionomycin for 6 h. Representative dot-plots of CD44 vs. **(A)** IL-17□ or IFN□ and **(B)** IL-4, IL-5, IL-13, IL17□, and IFN□ on gated CD3$^+$CD4$^+$ T cells. Numbers indicate the percentage of cells in the respective cytokine-producing gate. Data are representative of 3 independent experiments.

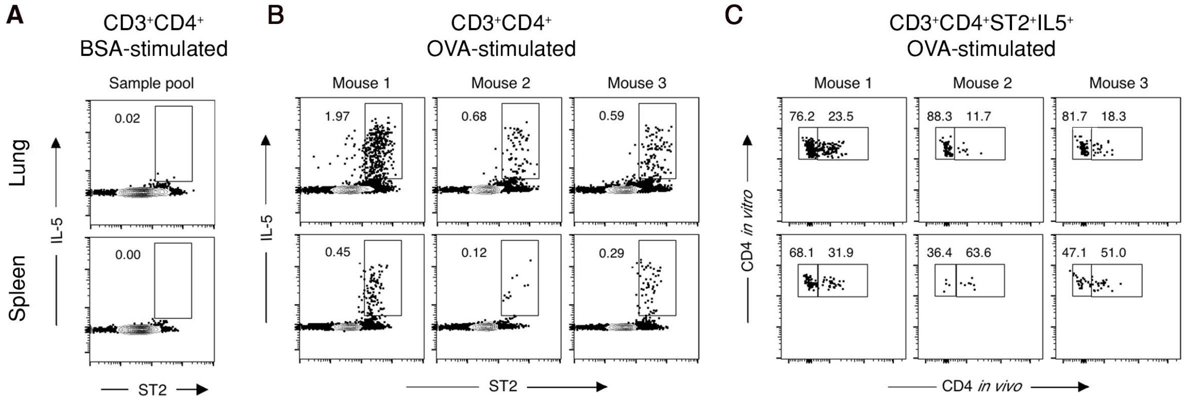

Supplementary Figure 7 | Protected lung and spleen Th2 cells from recovered mice on D665 produce IL-5. Anti-CD4 mAb clone RM4-4 was administered i.v. and 10-15 min later organs were extracted and lung and spleen cell suspensions from 3 recovered mice/group (from D665) were prepared and stimulated for 9 h with BSA- or OVA-loaded peritoneal macrophages and then stained with a non-overlapping anti-CD4 mAb (clone RM4-5), intracellular and cell surface mAbs. **(A)** Contour plots of ST2 vs. IL-5 from pooled lung and spleen cells, **(B)** Contour plots of ST2 vs. IL-5 from lung and spleen cells from individual mice, **(C)** Dot-plots of labeled vs. protected IL-5-producing $CD3^{+}CD4^{+}ST2^{+}$ T cells. Numbers indicate the percentage of cells in the respective cytokine-producing gate.

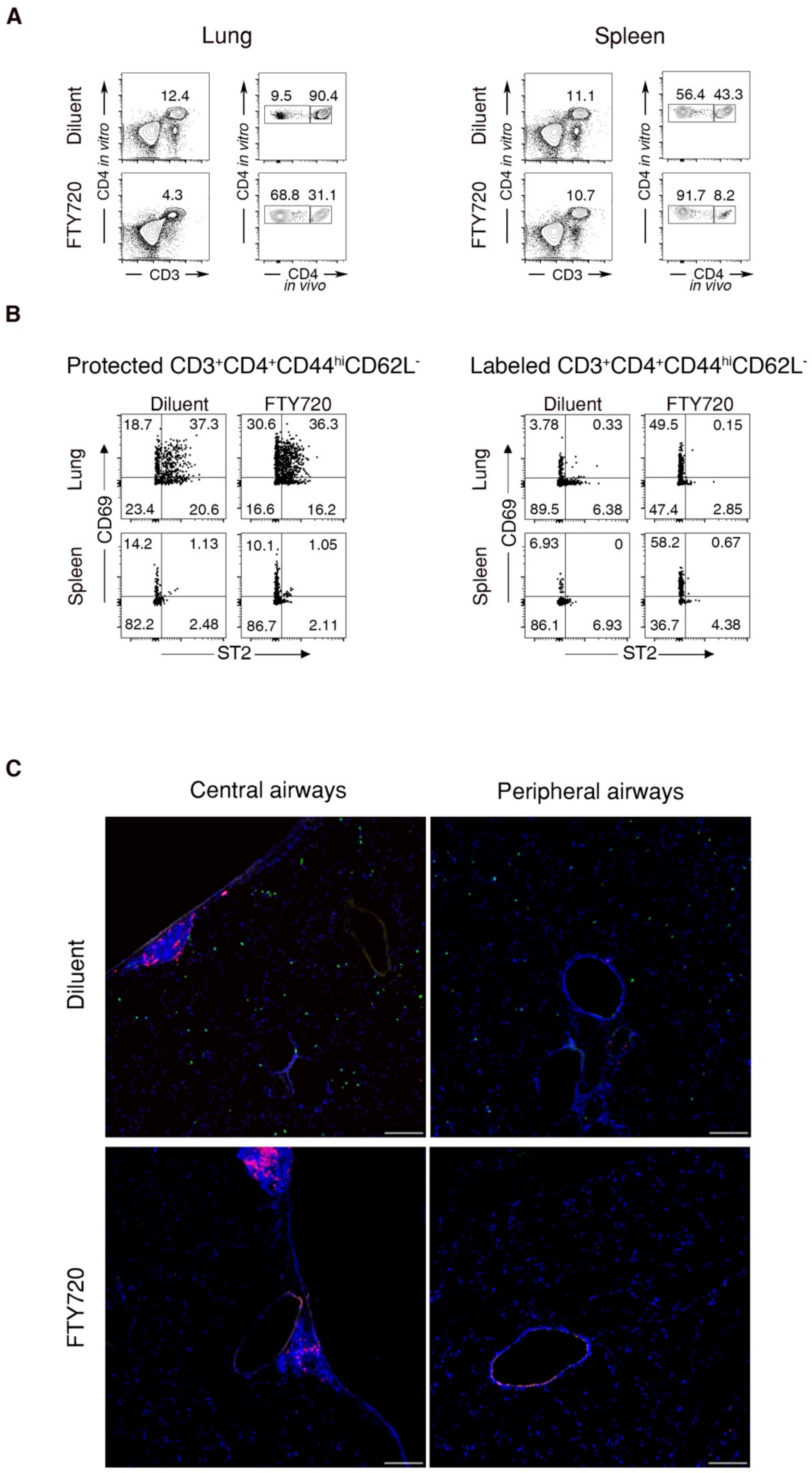

Supplemental Figure 8 | Protected CD3$^+$CD4$^+$ T cells remain in the lungs of recovered mice after FTY720 treatment. Recovered mice at D172 and D174 were treated with FTY720 (0.5mg/kg) or

diluent i.p. daily for 3 consecutive days. One day later, anti-CD4 mAb clone RM4-4 was administered i.v. and 10-15 min later organs were extracted and cell suspensions prepared, and stained with a non-overlapping anti-CD4 mAb (clone RM4-5) and antibodies for other extracellular markers for FACS analysis. **(A)** Representative contour-plots of total, labeled, and protected lung and spleen $CD3^{+}CD4^{+}$ T cells. **(B)** Representative dot-plots of CD69 and ST2 expression on protected and labeled $CD3^{+}CD4^{+}CD44^{hi}CD62L^{-}$ T cells. **(C)** Mice were administered with APC-labeled anti-CD4 mAb clone GK1.5 i.v., 10-15 min later, lungs were resected and embedded in OCT and then stained with Alexa Fluor 594-labeled anti-CD4 mAb clone GK1.5. Protected Th cells (red), labeled Th cells (green), tissue autofluorescence (yellow), and nuclear staining with DAPI (blue). Representative images of 5 mice/group. FACS plots are representative of 2 independent experiments.

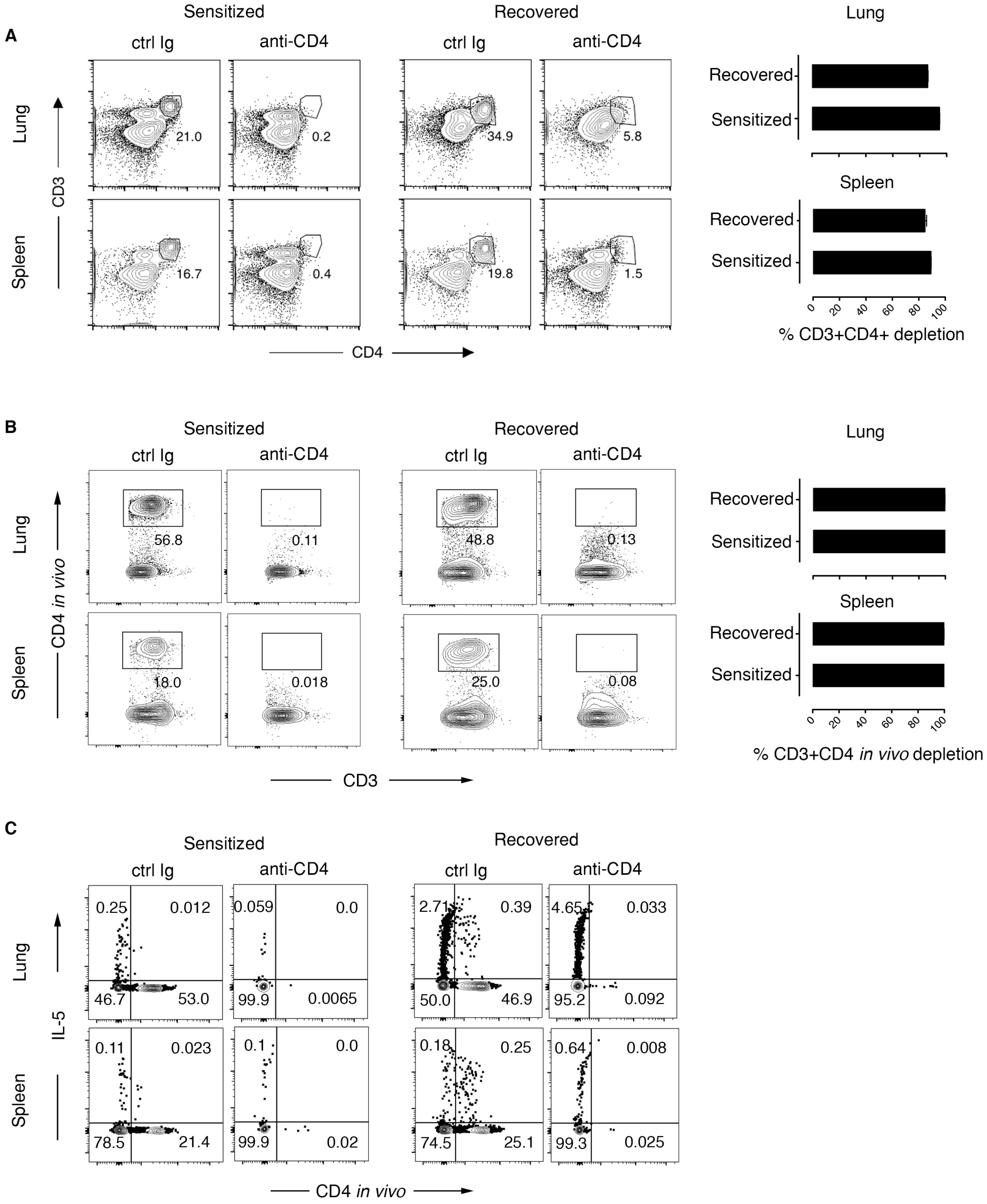

Supplementary Figure 9 | Anti-CD4 mAb depletes lung CD4$^+$ T cells more effectively in sensitized mice than recovered. Sensitized and recovered mice were treated i.p. with 0.2 mg of anti-CD4 depleting mAb (clone GK1.5) or isotype control antibody for 3 consecutive days. Three days after the last treatment, single lung and spleen cell suspensions were prepared for FACS analysis. **(A)** Representative contour-plots and percentage of depleted CD3$^+$CD4$^+$ T cells in lung and spleen of recovered and sensitized mice. **(B)** Representative contour-plots and percentage of CD3$^+$CD4$^+$ *in vivo*-labeled T cells. **(C)** Representative contour-plots of gated CD3$^+$ T cells stained with IL-5 and *in vivo* CD4.

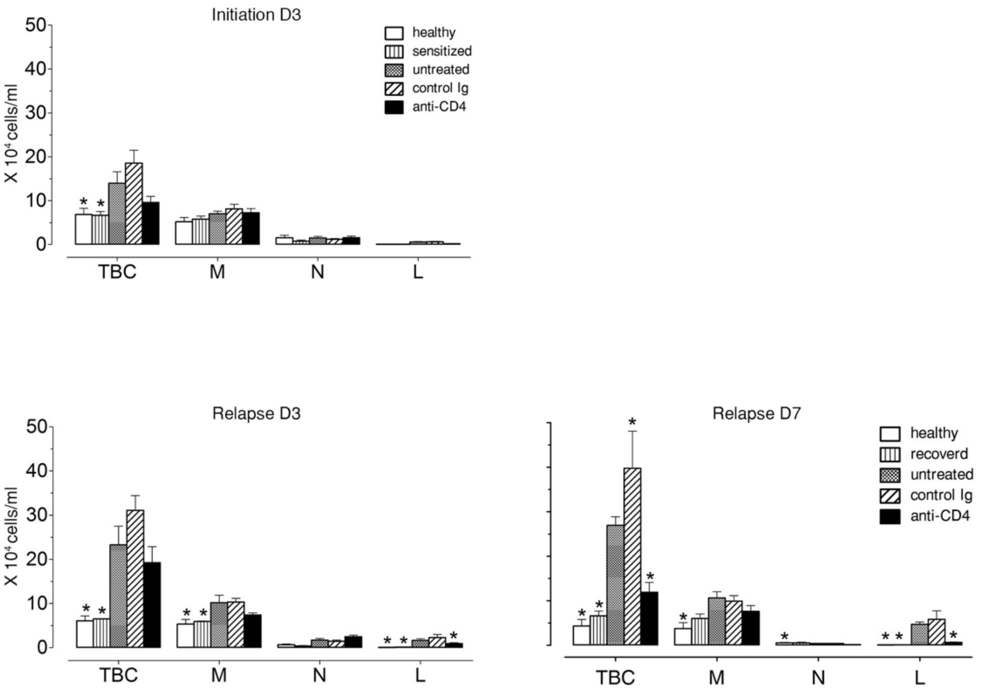

Supplementary Figure 10 | Sensitized and recovered mice were treated i.p. with 0.2 mg of anti-CD4 depleting mAb (clone GK1.5) or isotype control antibody for 3 consecutive days. Three days after the last treatment, mice were challenged with 100 µg of OVA i.n. to induce allergic asthma in sensitized mice or disease relapse in recovered mice. Graphs show total BAL cell numbers (TBC), and differential cell counts for macrophages (M), neutrophils (N), lymphocytes (L) at initiation, D3 and D7 after rechallenge. Data are presented as mean ± SEM from 2 independent experiments (*n*=*6*-*8*). $^{*}p < 0.05$ is statistically significant compared with the untreated group.

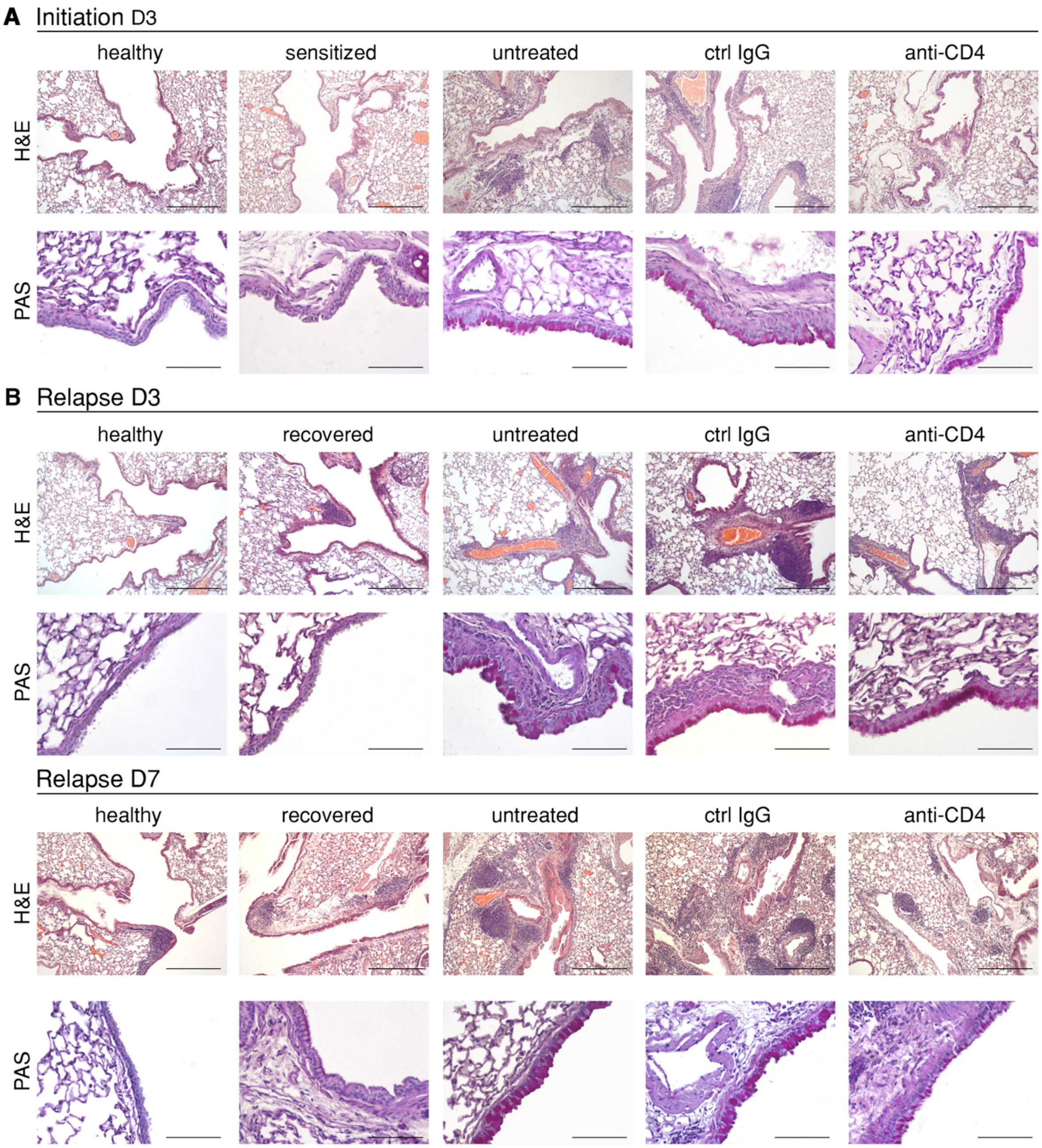

Supplementary Figure 11 | Lung inflammation and mucus hypersecretion are induced in response to allergen rechallenge after anti-CD4 mAb in recovered mice. Sensitized and recovered mice were treated i.p. with 0.2 mg of anti-CD4 depleting mAb (clone GK1.5) or isotype control antibody for 3 consecutive days. Three days after the last treatment, mice were challenged with 100 µg of OVA i.n. to induce allergic asthma in sensitized mice or disease relapse in recovered mice. Lung inflammation and mucus hypersecretion were evaluated D3 and D7 after allergen challenge. Representative photomicrographs of H&E- (scale bar 400 µm) and PAS-stained lung sections (scale bar 100 µm) **(A)** during disease initiation in sensitized mice and **(B)** at D3 and D7 after allergen rechallenge in recovered mice.
